# Serum S100B Levels and Major Depressive Disorder: Its Characteristics and Role in Antidepressant Response

**DOI:** 10.4306/pi.2008.5.3.193

**Published:** 2008-09-30

**Authors:** Byong-Su Jang, Hyeran Kim, Shinn-Won Lim, Ki-Won Jang, Doh-Kwan Kim

**Affiliations:** 1 Department of Psychiatry, Samsung Medical Center, Sungkyunkwan University School of Medicine, Seoul, Korea.; 2 Center for Clinical Research, Samsung Biomedical Research Institute, Seoul, Korea.

**Keywords:** S100B, Major depressive disorder, Antidepressant, Response, Neuroplasticity

## Abstract

**Objective:**

S100B is a neurotrophic factor that is involved in neuroplasticity. Neuroplasticity is disrupted in depression; however, treatment with antidepressants can restore neuroplasticity. S100B has previously been used as a biological marker for neuropathology and neuroplasticity; therefore, in this study, we compared serum S100B levels in depressive patients to those of normal controls. In addition, we compared the serum S100B levels of antidepressant responders to those of nonresponders.

**Methods:**

Thirty five normal controls and 59 depressive patients were enrolled in this study. Depressive patients entered a 6 week clinical trial that included treatment with antidepressants. The serum S100B levels and clinical assessments, which included Hamilton depression rating scores, were measured at baseline and after 6 weeks of treatment with antidepressants. The difference in the serum S100B levels between depressive patients and normal controls and between antidepressant responders and nonresponders was then compared.

**Results:**

There were no significant differences in the serum S100B levels of normal controls and depressive patients. In addition, 30 of the depressive patients responded to antidepressant treatment while 29 did not. Finally, the responders had significantly higher baseline serum S100B levels than the nonresponders.

**Conclusion:**

The results of this study suggest that the baseline serum S100B level is associated with the subsequent response to antidepressants. In addition, the high baseline serum S100B level that was observed in depressive patients may enhance neuroplasticity, which results in a favorable therapeutic response to antidepressants.

## Introduction

Several recent studies have reported that a change in neuroplasticity is associated with the pathophysiology of depression. In depressive patients, the serum levels of growth factors are decreased,^1^ and neuroplasticity, a fundamental mechanism of neuronal adaptation, is disrupted. In addition, recovery from depression is also associated with neuroplasticity,^2^-^4^ and the time lag that occurs between the administration of antidepressants and the onset of therapeutic activity suggests that the therapeutic effect of antidepressants arises from enhanced neurogenesis and the induction of neuroplasticity.^2^-^9^

S100B, which is one of the proteins involved in neuroplasticity,^10^,^11^ is located in glial cells in the human brain.^12^ SB100B is produced and secreted by astrocytes, and exerts paracrine and autocrine effects on neurons and glia.^13^ In addition, S100B is an acidic Ca^2+^ binding protein that may influence several cellular responses along the Ca^2+^-signal transduction pathway.^10^,^13^,^14^ Additionally, S100B regulates cell shape, contraction, cell-to-cell communication, energy metabolism, intracellular signal transduction, and cell growth.^15^ Cell culture and animal experiments have shown that the effects of extracellular S100B depend on its concentration.^10^,^16^ For example, when it is present in nanomolar concentrations, S100B acts as a growth and differentiation factor for neurons and astrocytes. However, when it is present in micromolar concentrations, S100B exerts neurotoxic activity and induces the apoptosis of neurons and astrocytes.

Several studies have reported that depressive patients have increased levels of S100B in the serum and cerebrospinal fluid (CSF).^17^-^20^ For example, Rothermundt et al. reported that the serum S100B levels in depressive patients were higher than those in normal controls, and suggested that a high level of serum S100B might correspond to neuron growth and synaptogenesis during synaptic remodeling in depressive patients.^17^ Additionally, Arolt et al. reported that antidepressant responders had higher baseline serum S100B levels than nonresponders, and suggested that high serum S100B levels were related to a favorable therapeutic response.^19^ The results of these previous studies suggest that S100B participates in neuroplasticity, which is important in recovery from depression. Furthermore, these findings indicate that the serum S100B level can be used to predict an antidepressant treatment response.

In this study, we compared the serum S100B levels of depressive patients to those of normal controls. We also assessed the serum S100B levels of depressive patients before and after treatment with antidepressants and then compared the serum S100B levels of antidepressant responders to those of nonresponders.

## Methods

### Subjects

Fifty-nine depressive patients that participated in the Clinical Trials Program of the Samsung Medical Center Affective Disorder Clinic were enrolled in this study. Patients received a semistructured diagnostic interview^21^ using the Korean version of the Diagnostic and Statistical Manual of Mental Disorders, Fourth Edition (DSM-IV),^22^ and all patients fulfilled the DSM-IV criteria for major depressive disorder. Diagnoses were confirmed by a board certified psychiatrist. Exclusion criteria for this study included significant medical conditions, schizophrenia or other psychotic disorders, bipolar disorder, a history of alcohol or drug dependence, epilepsy, and organic mental disorders. All patients enrolled in this study were at least 18 years of age.

Normal controls were recruited by advertisement in local newspapers. Normal controls met the following criteria: a Beck depression inventory (BDI)^23^ score of less than 16; a score on the Korean version of the mini mental status exam (K-MMSE)^24^ within the normal limits for their age and education level; a 7 Minute Screening battery score^25^ within normal limits for their age and education level; a Seoul-Activities of Daily Living (S-ADL)^26^ score of 0, and a Seoul-Inventory Activities of Daily Living (SIADL)^26^ score that was less than 8; no abnormal neurological signs. Normal controls were excluded based on the Healthy Screening Exclusion Criteria.^27^ Thirty five normal healthy controls were enrolled in this study.

The study protocol was approved by the institutional review board of Samsung Medical Center, Seoul, Korea. Signed informed consent was obtained from all participants.

### Procedure

We obtained blood samples by venipuncture and then measured the serum S100B levels. In depressive patients, blood samples were obtained prior to treatment with antidepressants (baseline S100B) and after 6 weeks of treatment with antidepressants (S100B after treatment).

The severity of the depressive symptoms was rated using the 17-item Hamilton Rating Scale for Depression (HAM-D),^23^ which was administered by a single trained rater. The 17-item HAM-D scores were obtained at baseline and after 6 weeks of antidepressant treatment. A response was defined as a decrease in the HAM-D score of 50% or more at 6 weeks.

The rater and laboratory workers were blind to the purpose of the study. In addition, the rater was blind to the S-100B data, and the HAM-D scores were not disclosed to the laboratory workers. To maintain blindness, a trained research coordinator managed all data and schedules.

The clinician's drug choice was based on the anticipated side effects and the symptomatic characteristics of the patients. The dosing protocol was flexible and conducted according to the clinician's assessment of symptoms and side effects. Side effects were measured using the UKU Side Effect Rating Scale^28^ at each visit. Anxiolytics and sedative-hypnotics were permitted.

### Biochemical assay

Venous blood was drawn using a serum-separating tube (SST) vacutainer, after which it was allowed to stand at room temperature for 30 min. The samples were then centrifuged at 3,000 rpm for 15 min, after which the serum supernatant was collected and frozen at -80℃ until assay. Qutantitation of the S100B serum levels was conducted using a CanAg S100 Enzyme immunometric assay kit (CanAg Diagnostics AB, Sweden) according to the manufacturer's instructions. This solid-phase, two-step, non-competitive immunoassay kit is based on two mouse monoclonal antibodies specific for two different epitopes expressed in S100B (S100A1B+S100BB). Briefly, calibrators and samples were incubated together with biotinylated Anti-S100B monoclonal antibody in Streptavidin coated microtiter strips. S100B present in the calibrators or samples was then adsorbed to the Streptavidin coated microtiter wells by the biotinylated anti-S100B monoclonal antibody during incubation. The strips were then washed and incubated with horseradish peroxidase (HRP) labeled Anti-S100B monoclonal antibody. Next, buffered Substrate/Chromogen reagent (hydrogen peroxide and 3, 3', 5, 5' tetra-methylbenzidine) was added to each well, which resulted in the enzyme reaction developing a blue color if antigen was present. After adding stop solution, the absorbance values were gotten at 405 nm. All analyses were conducted in duplicate in a double-blind test using an electronic pipetter. The lower detection limit of the assay for S100 was 25 ng/L. The imprecision of the intraassay (within-run CVs) was 1.1% at 0.054 ug/L, 4.6% at 0.489 ug/L, and 0.8% at 1.5 ug/L. The total imprecision (between-day, CVs) was 10.5% at 0.054 ug/L, 7.6% at 0.490 ug/L, and 0.8% at 1.5 ug/L.

### Data analysis

The means and standard deviations (SDs), ranges of continuous variables, and proportions of categorical variables were determined. Specifically, a T-test was used to evaluate continuous variables and a χ^2^ test was used for categorical variables. Accordingly, we compared the serum S100B levels of the depressive patients and normal controls using a T-test. Similarly, we compared the serum S100B levels of antidepressant responders and nonresponders at baseline and after treatment using a T-test. We then used multiple logistic regression analysis to determine if the S100B levels were different between antidepressant responders and nonresponders after adjusting for sex, age, body mass index (BMI, weight/height^2^), family history, episode numbers, onset age, and the duration of the current episode. In this analysis, treatment response was considered to be a dependent variable. All differences were considered to be significant at p<0.05.

## Results

### Subject characteristics

Of the 35 normal controls, 24 (69%) were female, and of the 59 depressive patients, 43 (73%) were female. The mean age of the normal controls was 61.8, and the mean age of the depressive patients was 60.3. The mean BMI of the normal healthy controls was 24.9 kg/m^2^ and the mean age of the depressive patients was 24.1 kg/m^2^. There were no significant differences in the mean sex ratio, age, or BMI of the depressive patients and the normal controls (Table 1).

A total of 59 depressive patients received antidepressants. Twenty two patients received mirtazapine, 29 patients received selective serotonin reuptake inhibitors (SSRIs)(fluoxetine, n=21; paroxetine, n=4; sertraline, n=3; and escitalopram, n=1), 3 patients received venlafaxine, 2 patients received nortriptylline, 1 patient received bupropion, and 2 patients received agomelatine. Of the 59 depressive patients, 30 were responders and 29 patients were nonresponders. Of the 30 responders, 14 received mirtazapine, 12 received SSRIs (fluoxetine, n=9; paroxetine, n=2; and sertraline, n=1), 1 received nortriptylline, 1 received bupropion, and 2 received agomelatine. Of the 29 nonresponders, 8 received mirtazapine, 17 received SSRIs (fluoxetine, n=12; paroxetine, n=2; sertraline, n=2; and escitalopram, n=1), 3 received venlafaxine, and 1 received nortriptylline. There were no differences in the mean sex ratio, age, BMI, family history, number of episodes, onset age, or duration of current episode between the responders and nonresponders (Table 1).

### Serum S100B levels

The mean serum S100B level of the depressive patients was lower than that of the normal controls, but this difference was not statistically significant (64.1±20.4 ng/L and 69.6±16.4 ng/L, mean±SD for depressive patients and normal controls, respectively, p=0.111).

**FIGURE 1:**
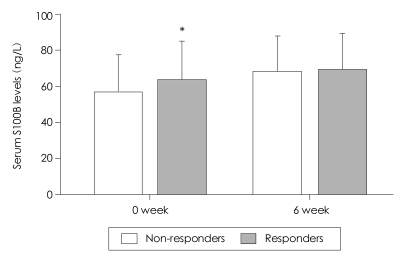
Comparison of serum S100B levels between antidepressant responders and nonresponders at baseline and after 6 week antidepressant treatment. Boxes represent means and error bars represent standard deviations. Serum S100B level was significantly higher in responders than in nonresponders at baseline. ^*^p<0.05.

When the serum S100B levels of antidepressant responders and nonresponders were compared at baseline, the S100B level of the responders was significantly higher than that of the nonresponders (68.6 ng/L for responders and 57.4 ng/L for nonresponders, p=0.048, Figure 1). After adjusting for sex, age, BMI, family history, number of episodes, onset age, and duration of the current episode, the baseline serum S100B levels of the responders were significantly higher than those of the nonresponders (logistic regression coefficient=0.05, χ^2^=5.31, df=1, OR=1.05, 95% CI: 1.01-1.10, p=0.02).

After 6 weeks of antidepressant treatment, the serum S100B levels of the antidepressant responders and nonresponders did not differ significantly (69.8±19.5 ng/L and 64.0±21.2 ng/L, mean±SD for responders and nonresponders, respectively, p=0.421, Figure 1). However, the serum S100B level increased after treatment with antidepressants, and this increase was more prominent in nonresponders than in responders. Specifically, the mean serum S100B level of the non-responders increased by 6.6 ng/L (57.4 ng/L to 64.0 ng/L), but the mean serum S100B level of the responders only increased by 1.2 ng/L (68.6 ng/L to 69.8 ng/L).

## Discussion

The results of this study indicate that the serum S100B levels did not differ between depressive patients and normal controls. However, depressive patients with high baseline serum S100B levels responded better to treatment with antidepressants than those with low baseline serum S100B levels. In addition, the serum S100B level increased in response to treatment with antidepressants.

In the present study, antidepressant responders had higher baseline serum S100B levels than non-responders. There are two causes of increased serum S100B levels, brain damage,^29^ and functional secretion by astrocytes.^30^ Mathematical models suggest that serum S100B levels above 350 ng/L indicate brain damage.^31^ In our study, the serum S100B levels were lower than 350 ng/L, which indicates that the levels observed in this study reflect functional secretion by astrocytes. S100B is known to have neurotrophic effects when it is present in response to functional secretion.^13^,^32^ For example, S100B enhances the growth and differentiation of neurons and astrocytes.^10^,^11^,^33^ In addition, S100B induces neurogenesis, which is essential to the behavioral effects of antidepressants.^5^,^34^ The results of this study suggest that the baseline serum S100B level is associated with enhancement of the growth and differentiation of neurons, which results in a favorable therapeutic response to antidepressants.

The results of previous studies comparing the serum S100B levels of depressive patients and normal controls showed that depressive patients had higher serum S100B levels than normal controls.^17^,^19^,^20^,^35^ However, there was no difference in the serum S100B levels of depressive patients and normal controls observed in the present study. It is important to note that there were several differences between the patient populations of the present study and those of previously conducted studies. Specifically, the depressive patients enrolled in previous studies were inpatients that had mean HAM-D scores that were greater than 25.^17^,^19^,^20^,^35^ Conversely, in the present study, the patients were outpatients with a mean HAM-D score of 19. However, the results of a previous study revealed a positive correlation between the severity of depression and serum S100B levels in depressive patients.^35^ This finding is similar to the results of the present study, in which there was a positive correlation between the HAM-D scores and the serum S100B levels (r=0.364, p=0.005). Therefore, the difference in the results of the present study and previously conducted studies may stem from differences in the severity of depression in the patient populations.

Another explanation for these differences may be the heterogeneity of neurotrophic activity in depressive patients. Neuroplasticity is disrupted in depression.^3^ In response, the brain attempts to restore neuroplasticity by increasing the S100B levels in depressive patients.^19^ However, the compensatory mechanism varies among depressive patients.^19^ As a result, some depressive patients have high concentrations of S100B, while others have low concentrations. Overall, the results of the present study indicate that the S100B levels of the depressive patients did not differ significantly from those of the normal controls. Moreover, neurotrophic activity is an indicator of a compensatory response to disrupted neuroplasticity, which is related to antidepressant response.^36^,^37^ Antidepressants exert their effects by increasing neurogenesis and modulating the signaling pathways involved in neuroplasticity.^36^,^37^ Therefore, the levels of neuroplasticity in depressive patients can affect their response to antidepressants.^36^,^37^

We also found that the serum S100B level increased after antidepressant treatment. Antidepressants stimulate the secretion of neurotrophic factors, such as brain derived neurotrophic factor (BDNF).^38^ Antidepressants also enhance the secretion of S100B by astrocytes via serotonergic systems.^15^,^39^-^41^ Interestingly, in this study, the increase in serum S100B levels in response to treatment with antidepressants was more prominent in nonresponders than in responders. Furthermore, in the present study patients with high baseline serum S100B levels were clinically improved within 6 weeks of treatment with antidepressants, but the increases in their serum S100B levels during 6 weeks of antidepressant treatment were minimal. Additionally, patients with low baseline serum S100B levels were not improved clinically within 6 weeks of treatment with antidepressants, but their serum S100B levels were much higher after 6 weeks of antidepressant treatment. In contrast to our findings regarding the levels of S100B, BDNF is known to increase more in responders than in nonresponders following antidepressant treatment.^38^ These findings suggest that S100B and BDNF play different roles in the recovery of depression. One explanation is that S100B may be a prerequisite for neuroplastic changes that is required to improve depression, whereas BDNF may reflect the current state of recovery.^38^

There are several limitations to this study. First, various antidepressants were used in our study. However, all antidepressants exert an effect on neuroplasticity.^42^ Second, the mean age of the patients included in this study was in the 60s, which is higher than that of other studies. Third, our study was a short-term clinical trial that was only conducted for 6 weeks. Despite these limitations, this study is the largest serum S100B study performed in depressive patients to date and provides useful information regarding the relationship between serum S100B levels and depression. However, the results of this study need to be confirmed in a long-term depression study. In addition, further studies including younger depressive patients using only one type of antidepressant are also needed.

## Figures and Tables

**TABLE 1:**
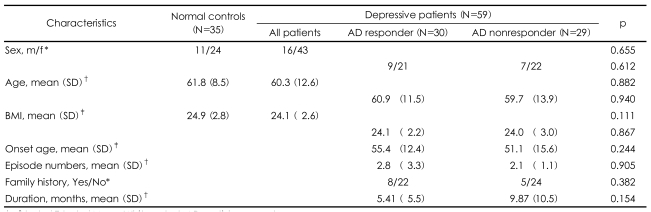
Demographic and clinical characteristics of study subjects

^*^χ^2^ test, ^†^T-test, ^‡^Mann-Whitney test. AD: antidepressant
